# A trip down memory lane with Retrovirology

**DOI:** 10.1186/s12977-019-0485-y

**Published:** 2019-08-21

**Authors:** Monsef Benkirane, Ben Berkhout, Persephone Borrow, Ariberto Fassati, Masahiro Fujii, J. Victor Garcia-Martinez, D. Margolis, Monique Nijhuis, Leslie Parent, Klaus Strebel, François Venter, Frank Kirchhoff, Andrew Lever, Susan Ross, Johnson Mak

**Affiliations:** 1 Centre National de la Recherche Scientifique, Montpellier, France; 2 University of Amsterdam, Amsterdam, The Netherlands; 3 University of Oxford, Oxford, UK; 4 University College London, London, UK; 5 Institute of Medicine and Dentistry, Niigata University, Niigata, Japan; 6 University of North Carolina, Chapel Hill, USA; 7 University Medical Center Utrecht, Utrecht, The Netherlands; 8 Pennsylvania State University, Hershey, USA; 9 NIAID, National Institutes of Health, Bethesda, USA; 10 Wits Reproductive Health and HIV Institute, University of the Witwatersrand, Johannesburg, South Africa; 11 University of Ulm, Ulm, Germany; 12 University of Cambridge, Cambridge, UK; 13 University of Illinois at Chicago, Chicago, USA; 14 Institute for Glycomics, Griffith University, Gold Coast, Australia

Fifteen years ago, *Retrovirology* was amongst the first open-access journals to be established through *Biomed Central*, instigated by our late Founding Editor Dr. Kuan-Teh Jeang. Since then, in what seemed like a rather daring move to be paper-free, *Retrovirology* has witnessed the exponential growth of open access journals that have changed the landscape of scientific publishing and communications. As was pointed out by the staff editors in PLoS Biology [1], the infancy of open access journal was a very different time from our present day, which was before smartphones, prior to most of the digital social media platforms and at a time when we had only begun to learn about the completed DNA sequences from the Human Genome Project [1].

Dr. Kuan-Teh Jeang (known universally as Teh) was an accomplished virologist and chief of the molecular virology section of the Laboratory of Molecular Microbiology at the NIAID NIH intramural program. Teh was a prolific scientist who authored more than 300 publications before he left us at the tragically young age of 54. Through open access publishing, Teh saw an opportunity to create a platform for retrovirologists to publish their findings in a timely manner as well as actively disseminating these publications to the community. Many of us vividly remember the enthusiastic emails that Teh sent us regarding any news or hot-off the press publications from *Retrovirology*.

From the inaugural digital issue of *Retrovirology*, Teh pledged to serve our community by providing fast, fair, and responsive editing to all submissions [2], a philosophy that remains as the cornerstone of our practice in publishing with *Retrovirology*. Since day one, Teh amassed together an impressive team of Founding Associate Editors to lead this journal, including Mark Wainberg, Andrew Lever, Monsef Benkirane, Ben Berkhout, Masahiro Fujii, and Michael Lairmore. Throughout the years, a number of well-regarded researchers have also joined the team of Associate Editors and Guest Editors in *Retrovirology*, including Robin Weiss, Persephone Borrow, Ariberto Fassati, J. Victor Garcia-Martinez, Paul Gorry, David Margolis, Monique Nijhuis, Leslie Parent, Klaus Strebel, Francois Venter, Frank Kirchhoff, Marit van Gils, Alexander Pasternak, and Rogier Sanders.

In 2005, Teh garnered financial support to establish the *Retrovirology Prize* designed, not as a typical award for lifetime achievement to the most senior researchers, but aimed at mid-career scientists between the ages of 45 and 60—‘arguably the most productive denizens of science’ (as Teh might have put it) [3] or ‘the glum-looking bunch of scientists’ traversing their “midlife crises” (as Teh would have put it) [3]. Joking aside, the *Retrovirology Prize* has provided us with an opportunity to honour some of the amazing scientists who have contributed to our community at their ‘mid-career’ stages but who are not yet senior enough to receive the awards commonly given to reflect the end of a career. As one would expect, the *Retrovirology Prize* has since become a highly sought-after award within our community, and many legends of our time are now part of this unique club of accomplished scientists (https://www.biomedcentral.com/collections/retrovirology-prize). The annual deliberation process is often fierce but respectful, and the only constant being that there are more deserving individuals than the single award that we are able to give out each year. In 2005, the first recipient of *Retrovirology* Prize was Professor Stephen Goff at Columbia University [4], and recipients of subsequent *Retrovirology Prize* winners are listed (https://www.biomedcentral.com/collections/retrovirology-prize). In recognition of the contribution of Teh to *Retrovirology* and to commemorate his untimely departure, the *Retrovirology Prize* was renamed as the *Kuan*-*Teh Jeang Retrovirology* Prize in 2015.

Throughout its short 15 years of existence, *Retrovirology* has grown from a modest journal that was largely driven by Teh, to a point where it is now recognised as a highly respected journal within our community. *Retrovirology* has witnessed and published a number of manuscripts that have had profound impacts on our field. While it is impractical to list or to recap all of the landmark works that have been published in *Retrovirology*, we have selected a few of our collative personal favourites to showcase some of the contributions that our colleagues have made through publications in *Retrovirology*.

Some of the most recognised publications include:

- Gila-Gaur and Strebel’s review on HIV Vif and APOBEC proteins [5];
- Descours et al. research manuscript on SAMHD1 HIV restriction in quiescent T cells [6];
- Sebastian and Luban’s research report on Trim5a restricts retrovirus via capsid domain [7];
- Weiss’s review on endogenous retroviruses [8];
- Towers’s review on tripartite motif proteins and cyclophilin A [9].

Other timely manuscripts include:

- Work dissecting the relationship between HIV transcription and latency by van Lint et al. [10];
- The series of manuscripts that helped to show that xenotropic murine leukemia virus related virus (XMRV) is not a human pathogen [11–23];
- An early study that helped set the tone of using humanised mice to study HIV infection and pathogenesis in vivo by Berges et al. [24]; and
- The potential roles of microRNA in retrovirus related diseases [25–28].

Other publication highlights can also be found in the following weblinks (incorporating all of the papers that team of AEs suggest).

Retrovirology has done many things like this well, but there are still many areas for us to improve on including widening the diversity of our publications. Traditionally, *Retrovirology* was set up as a journal with a strong molecular virology focus, and we have not published a significant number of manuscripts that have immunological-, clinical or structural biological themes. As we look toward our future, *Retrovirology* will actively support manuscripts in these areas of retrovirus research that have not been well represented in our publication history.

We also plan to increase diversity in our editorial board, authors and readers. In an attempt to better reflect the diversity of the retrovirus community, our current editorial board comprises researchers from most of the retrovirus research-intensive countries. As we don’t yet fully accurately reflect our community this is our aspiration and with the ongoing support from our submitting authors and readers, we are determined to make *Retrovirology* the journal that reflects the breadth of our collective values and commitment to our field.

With the sudden passing of Teh in 2013, Professors Mark Wainberg and Andrew Lever agreed to take on the roles of Co-Editor-in-Chief to lead our journal. Approaching the end of a 5 year term as Co-Editor-in-Chief for *Retrovirology*, Mark Wainberg sadly left us in an untimely manner in 2017 [29]. Mark had authored over 550 publication spanning his 30 years of work in retroviruses [29]. Andrew stepped down this year, becoming our first Editor emeritus. Retrovirology has survived all these ups and downs and with the support of the retroviral community we are beginning to see the metrics rise again.

With Andrew’s retirement as Co-Editor-in-Chief from Retrovirology, we are delighted to have one of our original commissioned meeting reviewers in 2004 [30], Professor Susan Ross, takes the helm as Co-Editor-in-Chief with Johnson Mak to serve our research community. We have also added 4 social media editors (Felipe Diaz-Griffero, Robert Gifford, Sallie Permar, and Kei Sato) to the board, who will be publicizing findings of interest to the Retrovirology community.

We have also been proud to support the bi-annual Frontiers in Retrovirology Conference, which has brought together scientists from all over the world to discuss state-of-the-art findings in the field. The upcoming Fall 2020 conference in Prague (to be organized and led by Jiri Hejnar) promises to continue the tradition of outstanding scientific exchange in the field.

In ancient Greece at around 500 B.C., Heraclitus of Ephesus coined the phrase ‘Change is the only constant’, *Retrovirology* is no exception to this rule. To remain relevant and important to our loyal scientific contributors the journal must evolve and adapt so that it continues to provide a unique specialist forum for this fascinating branch of science and medicine.
